# Utilization of Fibrous Mat Residues from Upholstered Furniture as Sustainable Fillers in Plywood Production

**DOI:** 10.3390/ma17164080

**Published:** 2024-08-16

**Authors:** Katarzyna Bartoszuk, Grzegorz Kowaluk

**Affiliations:** 1 Faculty of Wood Technology, Warsaw University of Life Sciences—SGGW, Nowoursynowska St. 159, 02-787 Warsaw, Poland; s207494@sggw.edu.pl; 2 Institute of Wood Science and Furniture, Warsaw University of Life Sciences—SGGW, Nowoursynowska St. 159, 02-776 Warsaw, Poland

**Keywords:** nonwoven fabric, plywood composite, mechanical properties, physical properties, recycling, upcycling, reinforcement, CCS

## Abstract

Nonwoven upholstery fabric is a waste product which is mainly generated during upholstered furniture production. The polyester composition makes it problematic to recycle and reuse this product. This study examined the manufacturing process of nonwoven fabric-reinforced plywood composites and their selected mechanical and physical properties. Nonwoven fabric was integrated between veneers bound with urea–formaldehyde resin to improve standard layered composites’ mechanical and physical properties. Several board variants were produced, differing in the position of the nonwoven layers in the composite structure. The composites were evaluated for modulus of rupture (MOR), modulus of elasticity (MOE), internal bond, and screw withdrawal resistance, among others. The results showed that the addition of nonwoven fabric significantly improved some properties, like internal bond and screw withdrawal resistance. Variants with strategically placed nonwoven layers showed the highest performance increases. The results underscore the potential of nonwoven fabric as an effective reinforcing material, offering a path to developing high-performance plywood composites suitable for demanding applications. Another environmental advantage is that the nonwoven fabric waste used in the tested plywood production has not been subjected to burning or landfilling but, through its incorporation into plywood structure, has positively contributed to the Carbon Capture and Storage (CCS) policy. The findings advocate for a circular economy approach, in which industrial waste is effectively repurposed, contributing to the development of green materials in the wood-based composite industry.

## 1. Introduction

Despite the development of many new wood materials, plywood is still valuable in many industries [1], including the construction, furniture, and automotive sectors, due to its favorable strength-to-weight ratio and versatile properties [2]. However, traditional plywood often faces mechanical strength, durability, and moisture resistance limitations, necessitating the development of enhanced composite materials [3]. A plywood composite consists of at least two layers of outer laminate, separated by an inner laminate [4]. The outer layers can be relatively thin, from about one millimeter, while the core layer can range from a few millimeters to several centimeters. Such composites are characterized by high strength properties, low specific weight, relatively low production costs, and high durability [5]. A big problem in plywood production is formaldehyde release, especially from urea–formaldehyde-bonded plywood [6]. The technology used in plywood production has not changed for over a century. However, research is constantly being conducted to improve its quality [7]. On a daily basis, the furniture industry uses adhesives with the addition of various fillers for plywood production. They improve the physical, mechanical, technological, and operational properties. Components are increasingly subject to modifications—veneers and adhesives but also fillers [8]. Veneer impregnation has also been explored to improve fire resistance, with the use of appropriate measures [9]. With increasing public awareness of and emphasis on eco-friendly behavior, the wood industry is forced to use more environmentally friendly practices. This has led to various attempts to replace formaldehyde-based thermosetting resins with natural-origin adhesives. For instance, glutaraldehyde-modified starch has been considered an alternative binder in plywood technology, with studies confirming its effectiveness [10]. Wood bark has also been used as a filler to minimize free formaldehyde emissions [3,11]. Green tea leaves have demonstrated a similar ability to reduce formaldehyde emissions when used as a filler [12].

There have also been attempts to modify plywood structure by adding fibrous materials. An example is carbon fibers added to five-layer plywood [13]: the bending properties were improved, but water uptake was worse for carbon fiber-modified plywood. The carbon fiber fabric was also successfully used by [14], in which case the poplar/eucalyptus composite plywood formwork was reinforced. Applying glass fiber woven fabric improved the modulus of rupture and modulus of elasticity of the plywood modified by these materials. Still, that improvement could also have resulted from the higher density of the tested layered composites [15]. Natural-origin fibers, like cellulose and flax fibers, have been used to modify plywood structure [16]. The research shows that the non-treated cellulose and flax-reinforced nonwoven fabrics have similar mechanical behaviors. The hydrophobic pretreatment of cellulose nonwoven fabrics improved the performance of plywood regarding tensile strength, shear strength, screw withdrawal resistance, and modulus of rupture. Still, it lowered the modulus of elasticity compared to the reference.

Integrating nonwoven fabrics into composite materials has enhanced their properties significantly. An essential aspect of using nonwoven fabric in producing fiberboards is its impact on the bonding process of these boards. Considering the production process of these materials, it may turn out that they are not chemically inert, and their characteristics can be variable. However, the Acid-Buffering Capacity (ABC) parameter can be applied in wood technology and may help adjust the production parameters if such a non-inert material causes issues [17]. Consequently, this does not pose a risk of necessitating the development of additional techniques and methods. Nonwoven fabrics, particularly nonwoven upholstery, are characterized by high porosity, flexibility, and ability to form interfacial solid bonds with other materials, making them suitable for reinforcing layered composites such as plywood [18,19,20,21]. Plywood composites, including nonwoven fabrics, can lead to better stress distribution and bonding between layers, enhancing overall performance [22].

Nonwoven fabrics have unique structural characteristics due to their manufacturing process, which involves bonding fibers together without weaving, knitting, or stitching. These fabrics offer several advantages, such as improved tensile strength, impact resistance, and dimensional stability, which are critical for enhancing the properties of plywood composites [23]. Specifically, needle-punched nonwoven fabrics have shown promise in reinforcing polymer composites due to their ability to distribute stress uniformly and provide better mechanical interlocking [24].

To investigate the potential of nonwoven fabric-reinforced plywood composites, this study focuses on manufacturing and testing several board variants with different positions of nonwoven layers. Their mechanical properties, including modulus of rupture (MOR), modulus of elasticity (MOE), and screw withdrawal resistance (SWR), were evaluated. Additionally, density and density profile measurements and thickness swelling and water absorption tests were conducted to assess the physical properties of the composites. Industrial layered composites were used as reference materials to benchmark the performance of the nonwoven reinforced composites.

## 2. Materials and Methods

### 2.1. Materials

This study concerned the production of five-layered plywood using rotary-cut birch veneers (Betula spp.). The veneers had a thickness of 1.8 mm, moisture content (MC) of approximately 6%, and dimensions of 300 mm × 300 mm.

The binder was industrial urea–formaldehyde (UF) resin Silekol S-123 (Silekol Sp. z o. o., Kędzierzyn-Koźle, Poland). Additionally, an ammonium nitrate water solution was used as a hardener when samples were subjected to a temperature of 100 °C to reach a curing time of about 86 s. In addition, rye flour (PPHU JS, Magnoliowa St. 2/11, 15-669 Białystok, Poland) was used as a bonding mass filler.

**Figure 1:**
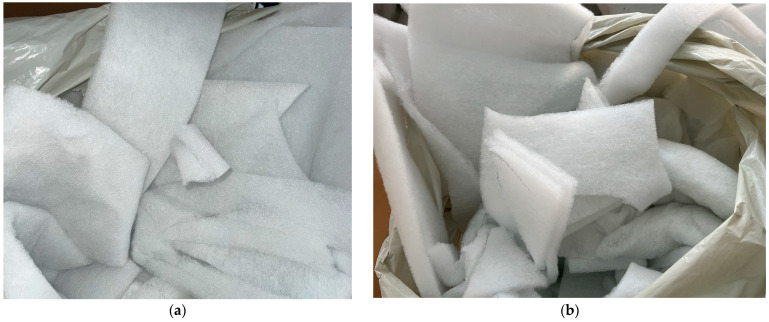
(**a**,**b**) Examples of the nonwoven fabric as a post-production waste of irregular shapes and dimensions from the production of upholstered furniture.

The nonwoven fabric used in the tests was from PPHU “ADAX” (Topola Szlachecka 21, 99-100 Łęczyca, Poland). It is a two-component polyester fiber with a low melting point. It is a white fiber; the available form is ca. 35 mm thick, with a 200 g m^−2^ grammage, and it is fire-resistant. The nonwoven fabric was a post-production waste of irregular shapes and dimensions from the production of upholstered furniture (Figure 1). The obtained pieces of various sizes were used to form 300 mm × 300 mm mats.

### 2.2. Preparation of Panels

A five-layer reference plywood glued with urea–formaldehyde (UF) resin with hardener, demineralized water, and flour filler was produced as part of this research. The adhesive mixture was prepared in parts by weight (pbw): 100:10:16:5 (resin/hardener/filler/water). A brush was applied to the adhesive mix spread to the veneers at 180 g m^−2^ per single bonding line. Whenever nonwoven fabric appeared between veneers, the glue mass was spread to both surfaces surrounding the fabric. Cut to shape/size was the only step of fabric preparation before application in plywood (no thickness reduction or milling). The veneers were stacked alternately and then pressed in a heated hydraulic press (AKE, Mariannelund, Sweden) for 7 min at a pressing temperature of 140 °C and a maximum unit pressing pressure of 1.2 MPa. Non-sticking, heat-resistant polytetrafluoroethylene (PTFE) mats were used to avoid sticking of the outer plywood layers to the press. After pressing, the samples were conditioned at 20 ± 1 °C and 65 ± 2% relative humidity (RH) for seven days to stabilize the mass before testing.

The amount and distribution of nonwoven layers differentiated the panels (Table 1). Five different variants were created with layers of nonwoven fabric at various locations. Reference panels (hereafter: REF) were also produced, and no nonwoven fabric was added to these.

### 2.3. Characterization of the Elaborated Panels

Mechanical tests were performed on a computer-controlled universal testing machine (Ośrodek Badawczo-Rozwojowy Przemysłu Płyt Drewnopochodnych Sp. z o.o., Czarna Woda, Poland). Modulus of rupture (MOR) and modulus of elasticity (MOE) tests were conducted in accordance with current standards [25]. In addition, a screw withdrawal resistance test [26] and an internal bond (IB) test [27] were completed. Using the test procedure specified in the standard for particleboard and fiberboard, the thickness swelling after immersion in water was determined, and analysis was carried out for all variants [28]. In addition, a water absorption test was conducted on the samples used for the thickness swelling test. Each test was performed in six repetitions. The density profile was also obtained for all variants (three replicates from each variant) using a Grecon DAX 5000 instrument (Fagus-GreCon Greten GmbH and Co. KG, Alfeld/Hannover, Germany), based on X-ray technology, with a sampling step of 0.02 mm and a measurement speed of 0.1 mm s^−1^. All the tests were completed under air conditions of 20 ± 2 °C and 65 ± 5% RH.

### 2.4. Statistical Analysis

Analysis of variance (ANOVA) and t-test calculations were conducted to identify significant differences (α = 0.05) between factors and levels when applicable, using the IBM SPSS statistic base (IBM, SPSS20, Armonk, NY, USA). The homogenous groups are indicated in Table 2. The results shown in the graphs represent mean values, and standard deviations are shown as error bars.

## 3. Results and Discussion

### 3.1. Modulus of Rupture and Modulus of Elasticity

**Figure 2:**
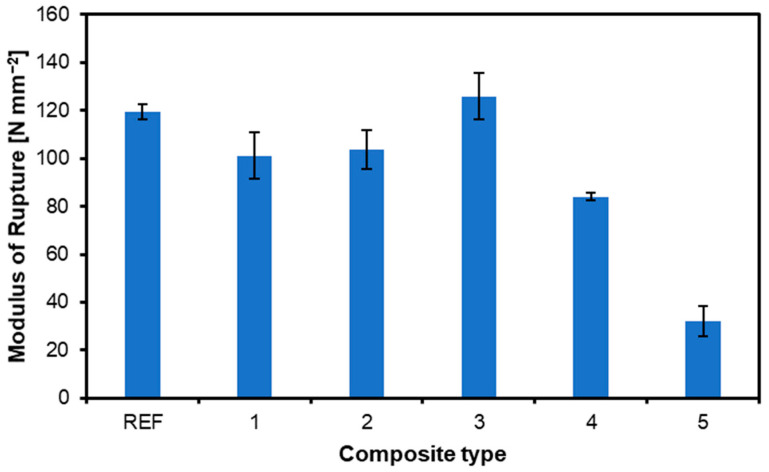
Influence of composite type on MOR of produced plywood.

**Figure 3:**
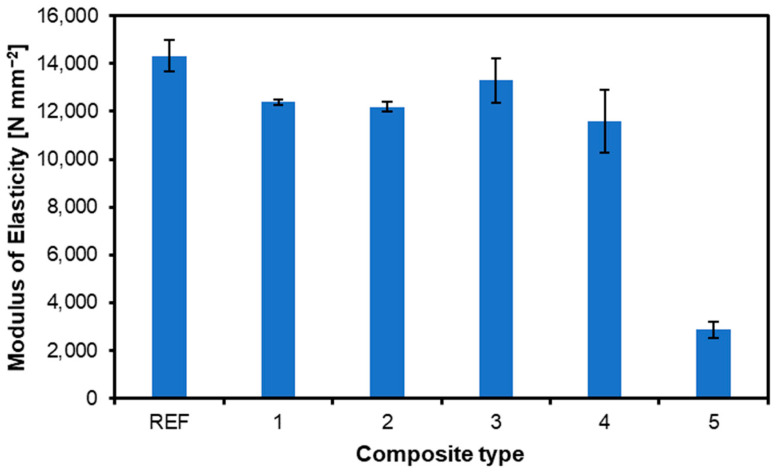
Influence of composite type on MOE of produced plywood.

Figure 2 and Figure 3 show the dependence of the modulus of rupture and modulus of elasticity on the content and location of nonwoven layers, respectively. It can be seen that the content of nonwoven layers does not drastically change the results, and surprisingly, in most cases, it even worsens them. For the reference sample, the MOR value is 119 N mm^−2^, and for variant 3 (nonwoven fabric mat located under face veneers only), it is 126 N mm^−2^. The lowest value was obtained for variant 5, with nonwoven fabric content. No rule showing a decreasing or increasing tendency in strength for the number of nonwoven fabric layers can be deduced here. Dasiewicz and Wronka [29] obtained a similar lack of dependence: the highest MOR was for the reference sample and the lowest was for the sample with 1% chestnut flour filler content. The location of the plywood-modifying layer in their structure shows that the best bending properties, like MOR and MOE, can be reached when the reinforcing material is located between the outer veneers [13]. However, in [14], the best bending properties were achieved with the reinforcing layer on the plywood surfaces and in the middle of the plywood thickness.

In the case of MOE (Figure 3), the reference sample achieved the best result without adding upholstery nonwoven fabric (14,308 N mm^−2^). The lowest MOE value again occurred for variant 5 (nonwoven content in the board), 2873 N mm^−2^, where the content of waste fabric was the highest. The only relationship when comparing the MOR and MOE results appears in the reference sample and the sample of variant 5, which have the highest and lowest results. However, analyzing the remaining variants with the addition of nonwoven layers, it can be concluded that variants 1–4 have very similar MOE scores (statistically homogeneous group). The MOE and MOR results, when compared to the standard requirements [30], show that the best-tested sample (composite type 3) reached the F 80 class (the top one, for above 120 N mm^−2^) for MOR and the highest class, E 140, for MOE (over 12,600 N mm^−2^). In light of this, composite 5, with the lowest parameters, achieves classes F 20 and E 30 for MOR and MOE, respectively. However, Bal and Bektas [31], comparing the properties of plywood made of poplar, eucalyptus, and beech, obtained increased flexibility after changing the plywood structure. These conclusions are consistent with the results of previous research by Biadała et al. [32].

### 3.2. Internal Bond Strength and Screw Withdrawal Resistance

**Figure 4:**
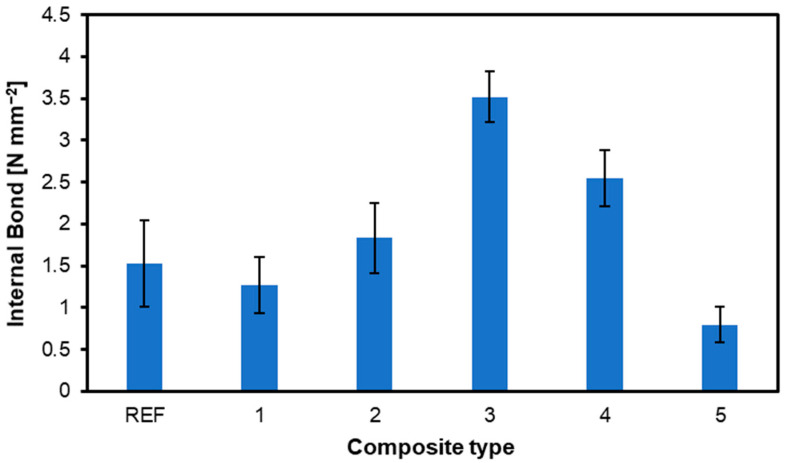
Internal bond of plywood with various content and location of nonwoven fabric.

**Figure 5:**
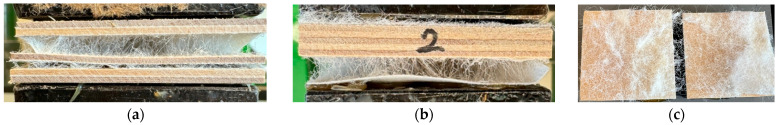
Examples of the destruction of the tested composites after the internal bond test: (**a**) plywood 1, (**b**) plywood 2, (**c**) the inner surface of the nonwoven fabric layer.

The results of internal bond strength measurements are shown in Figure 4. Internal bonding, according to EN 319 [27], is designed to test materials based on wood particles. It is considered a simple test and can be used to evaluate the perpendicular tensile strength of plywood [33]. As can be seen in the figure, the highest strength was obtained by the sample of the third type, which had two reinforcing layers of nonwoven fabric located between two face veneers, and its strength was 3.52 N mm^−2^. Such a location and the content of fabric can be the reason for its having the highest IB because the fibrous material, which can act as a thermal insulator during hot pressing, was heated well enough to allow for binder curing and proper densification of the layers due to its optimal location. The sample that obtained the second highest strength was the sample of the fourth type, which achieved 2.54 N mm^−2^ of internal bond strength. The lowest internal bond strength was obtained by the sample of the fifth type, with a strength of only 0.8 N mm^−2^. The lowest average values of panel no. 5 can be partially explained by the nonwoven fabric between all the veneers and the faces of the composite. Such a structure can influence the pressing process since the nonwoven fabric can act as a thermal insulation. However, separate research should be completed to verify that theory. Examples of the tested composites’ destruction after the internal bond test are presented in Figure 5. As can be seen, in the case of plywood 1, built with nonwoven fabric between every veneer, the destruction occurred in the core zone, away from the surface, in the nonwoven fabric structure. The fabric structure was also damaged during the IB test of panel no. 2, where the nonwoven fabric was present only on the surface of the outer veneers. The figure (Figure 5c) confirms the proper adhesion of the fabric to the veneers since the destruction that occurred during the IB test happened in the structure of the fabric. It is essential to mention the influence of preparation and testing on the test sample. First, the effect of temperature (ca. 200 °C) when gluing the samples to the aluminum metal block caused tension inside the surface layers due to sharp temperature differences. Second, the adhesive overlaps on the outside of the sample in the tensile direction. These are two potential errors in the test results, as mentioned, among other factors, by Bekir et al. (2015) [15]. To improve and gain a deeper understanding of the strain distribution under tensile loading, it is recommended to use digital image correlation (DIC) for analysis as a value addition method, according to Li et al. (2020) [34].

**Figure 6:**
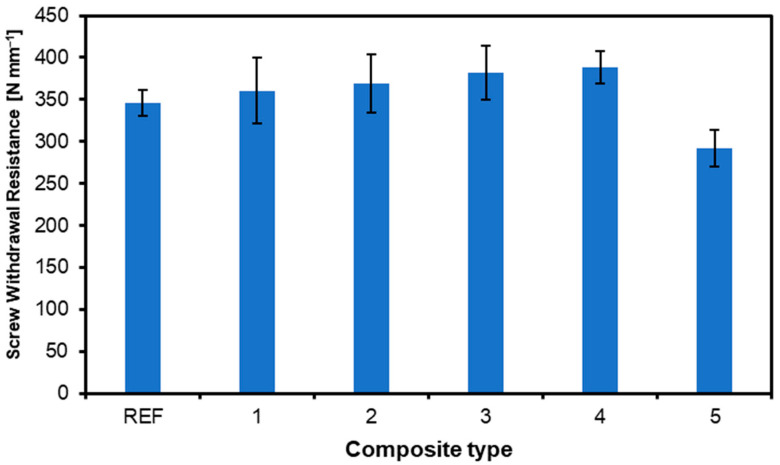
Screw withdrawal resistance of plywood with various contents and locations of nonwoven fabric.

**Figure 7:**
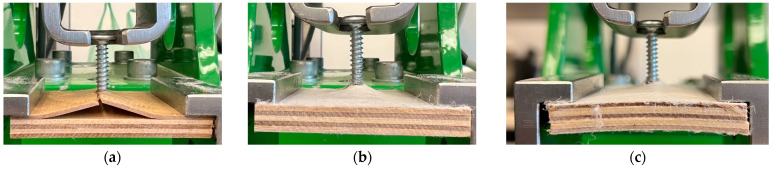
Examples of tested composites’ destruction after screw withdrawal resistance test: (**a**) plywood REF, (**b**) plywood 2, (**c**) plywood 5.

The main connecting element for wood-based materials, such as plywood or OSB, is screws. Therefore, screw withdrawal resistance is one of the critical factors for wood-based materials used in construction [35]. Figure 6 shows the average screw withdrawal resistances. The average resistance obtained for the reference board is 346 N mm^−2^. This is neither the highest nor the lowest value obtained for the tested samples. The highest value was obtained for the sample of variant 4, which amounted to 388 N mm^−2^. In contrast, the lowest value was again obtained for the variant 5 sample and was 292 N mm^−2^. Some pictures of the samples during the SWR test are presented in Figure 7. The reference sample, which reached one of the lowest average values for SWR, was damaged by delamination of the face veneer. In the case of plywood 2 (Figure 7b), for which the SWR was higher than for the REF panels, the withdrawing screw broke the composite structure in the screw location zone. The lowest SWR average values were registered for plywood 5, presented in Figure 7c. It is shown that the sample damage occurred due to delamination in the core zone. This remark is in line with the observations of the IB tests, where panel no. 5 has the lowest IB values.

According to [13], the location of the fibrous reinforcing layer can significantly influence the screw withdrawal resistance. In the mentioned research study, the best results for that feature were found when the reinforcing fibrous layers were placed in the core zone of the tested composite. This is in line with the results achieved for sample 4. The type of reinforcement provides a slight improvement for linen fabric compared to celluloses A and B. Analyzing the different fabrics, there is a slight trend where a larger amount of glue improves the screw withdrawal resistance. Pretreatment of the cellulose fabric affects this resistance. Increasing the amount of glue leads to higher density, which is consistent with the idea that density affects screw withdrawal resistance [36], in addition to other wood-related parameters such as fiber direction, grade, moisture content, and temperature [37]. Fiber reinforcement affects screw withdrawal resistance depending on the location of the reinforcing fabric in the board structure [38,39]. The influence of specific fabric characteristics requires further research [16].

### 3.3. Density Profile

**Figure 8:**
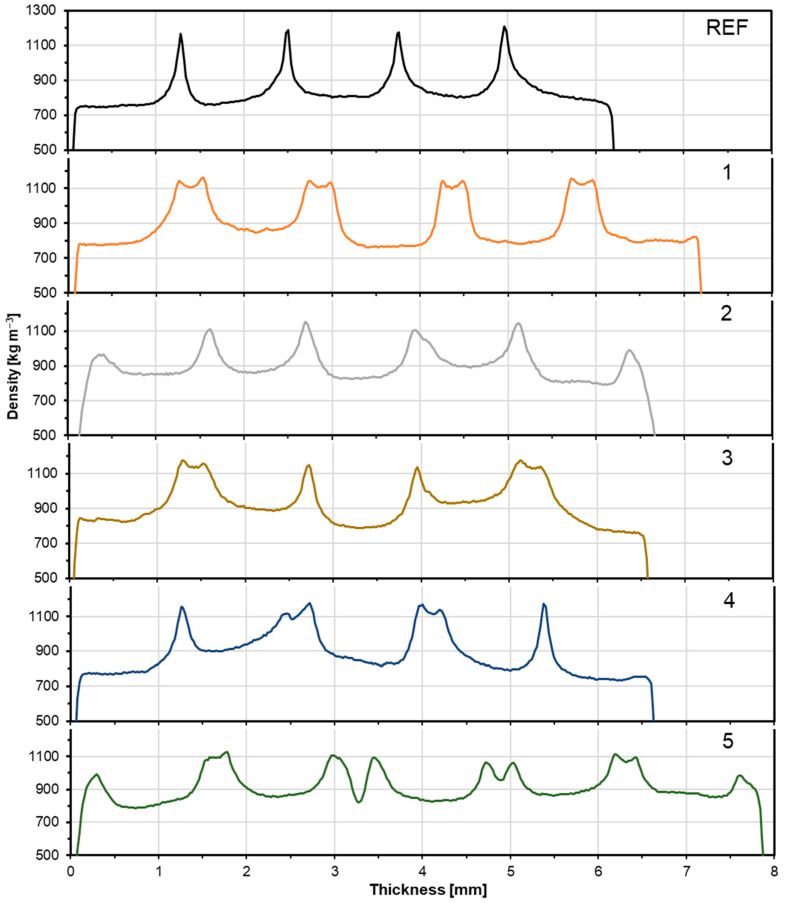
Density profile depending on the type of composite.

Figure 8 shows the density profiles of samples with layers of nonwoven upholstery fabric with a reference variant. The veneers showed a density of about 750–800 kg m^−3^, depending on the sample. The thickness and density of the nonwoven fabric were about 0.3 mm and 1100 kg m^−3^, respectively, and the reasons for that densification and thickness reduction were the binder impregnation and curing in the fabric structure during hot pressing. The bond line showed a bond density slightly above 1200 kg m^−3^ for the reference sample. The same result was obtained by Wronka and Kowaluk [8] in a study of upcycling wood dust from recycled particleboard as filler in lignocellulosic composite technology. A similar result was obtained by Daniłowska and Kowaluk [40] in an analysis of the use of coffee bean extraction residue as a filler in plywood technology. As for the samples reinforced with nonwoven upholstery fabric, all variants had a similar bond line density and were almost identical to the reference sample, which had a bond line density of about 1200 kg m^−3^. Differences in bond line densities within the same variant were also observed, which can be explained by excessively high viscosity during adhesive application, resulting in uneven bonding.

### 3.4. Thickness Swelling and Water Absorption

**Figure 9:**
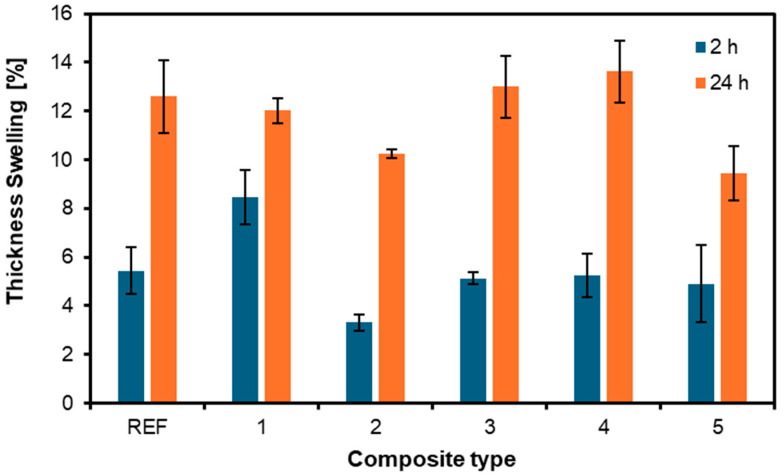
Thickness swelling of the panels produced with the use of different arrangements of nonwoven fabric layers.

**Figure 10:**
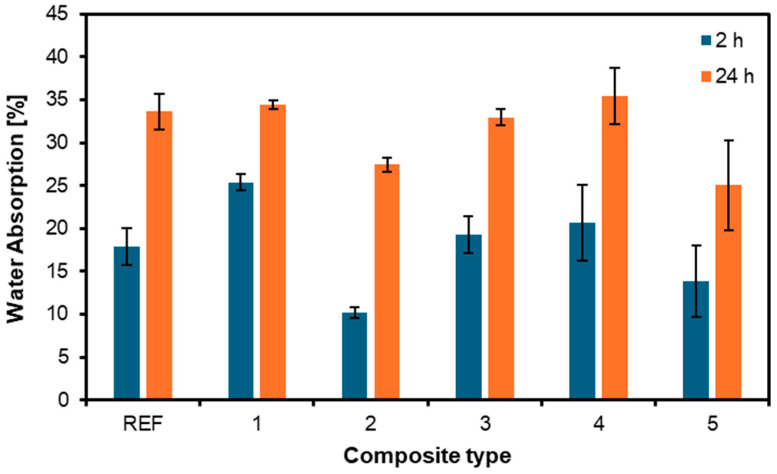
Water absorption of the panels produced using different arrangements of nonwoven fabric layers.

The results for thickness swelling and water absorption are shown in Figure 9 and Figure 10, respectively. After 24 h of soaking, the intensity of the thickness swelling was more pronounced in all samples than after 2 h. The lowest values after 2 h and 24 h were in the samples whose outer layers were nonwoven upholstery layers. This can only indicate the positive effect of the nonwoven fabric on the plywood and its reinforcing properties. After 2 h, the reference board had a swelling value of 5.4%; after 24 h, it was already 12.6%. The most significant difference in swelling per thickness after 2 and 24 h was in the variant 4 sample, which had nonwoven layers only inside the board. The swelling value after 2 h was 5.3%, and after 24 h, it was already 13.6%. The works of Buzo et al. [41] and Sugahara et al. [42] also found lower thickness swelling values for particleboards made of PU compared to ones made of formaldehyde-based resins.

The results for the water absorption of the tested panels with different arrangements of nonwoven fabric layers are shown in Figure 10. The values achieved after 24 h are very close for all types of samples, even the reference one. On the other hand, the samples’ values after two hours are more diverse. The variant 2 sample obtained the lowest value, 10.2%, and its value after 24 h was 27.5%. In contrast, the variant 1 sample obtained the highest value after 2 h, 25.4%, and its value after 24 h was 34.4%.

The differences among the TS and WA samples with different amounts and locations of nonwoven upholstery fabric waste can be attributed to the differences in the water wettability nature of polyester, the source fiber of the fabric, and of wood. According to [43], the water contact angle of polyester fabric can be about 143°, whereas the birch veneer contact angle is about 64° [44]. A study by Setter et al. [45] using different resins (phenolic and urea–formaldehyde) also showed a significant difference in water absorption. The authors concluded that the PF adhesive is traditionally used in panels intended for outdoor use because of its excellent moisture resistance.

## 4. Conclusions

The novelty of this research is the approach to using non-wood waste from the production of upholstered furniture in wood-based composites such as plywood. This work aimed to demonstrate the possibility of recycling upholstery nonwoven fabric waste by incorporating it into producing plywood panels. The results show that the location of nonwoven fabric on the outer layers has the most significant impact on the strength of plywood. Boards with a layer of nonwoven fabric added to the inner joints showed no substantial improvement in strength, and in some tests, even a decrease in strength was observed. Instead, an inverse relationship can be seen in the internal bond and screw withdrawal resistance tests, where higher strengths were obtained by variants with the addition of nonwoven fabric in the inner layers. Nonwoven layers do not significantly affect the density profile of the panels. The densities of both the bond with adhesive alone and the bond with the addition of nonwoven fabric are very similar. Considering the obtained results, the potential application scenario for the tested plywood can be, for example, structural sheathing in wooden frame buildings, where the proper internal bond and screw withdrawal resistance are the requested features in light of further fixing of screws or load application on walls or roofs.

In summary, it can be concluded that upholstery nonwoven fabric is a promising additive for reinforcing plywood, especially in the outer layers. Such use is a promising result in the context of a circular economy and waste recycling principles. The use of waste from the production of upholstered furniture will, at a minimum, help solve the problem of the amount of waste generated in this industry and its management. Further research on plywood with nonwoven fabric waste from upholstery furniture can be directed to evaluate its long-term properties under various conditions and optimize the type and amount of binder applied.

## Figures and Tables

**Table 1:** Codes and structures of produced composites.

Composite Code	Structure of the Produced Plywood
REF	V ^1^ V V V V
1	V W V W V W V W V
2	W V V V V V W
3	V W V V V W V
4	V V W V W V V
5	W V W V W V W V W V W

^1^ V—veneer; W—nonwoven fabric.

**Table 2:** Statistical assessment results of mean values.

Test Type	Type of the Tested Panel					
	REF	1	2	3	4	5
MOE	a ^1^	b	b	a, b	b	c
MOR	a	b	b	a	c	d
IB	a	a	a	b	c	d
SWR	a	a	a	a	b	c
TS 2h	a	b	c	a	a	a
TS 24h	a	a	b	a	a	b
WA 2h	a	b	c	a	a	a
WA 24h	a	a	b	a	a	b

^1^ a, b… homogeneous group.

## Data Availability

The data presented in this study are available in the open-access repository: https://doi.org/10.18150/8Z9LKM.

## References

[1] Dyrwal P., Borysiuk P. (2020). Impact of Phenol Film Grammage on Selected Mechanical Properties of Plywood. Ann. WULS For. Wood Technol..

[2] Alam M.A., Yadama V., Cofer W.F., Englund K.R. (2014). Analysis and Evaluation of a Fruit Bin for Apples. J. Food Sci. Technol..

[3] Mirski R., Kawalerczyk J., Dziurka D., Siuda J., Wieruszewski M. (2020). The Application of Oak Bark Powder as a Filler for Melamine-Urea-Formaldehyde Adhesive in Plywood Manufacturing. Forests.

[4] Burawska-Kupniewska I., Borowski M. (2021). Selected Mechanical Properties of the Reinforced Layered Composites. Ann. WULS For. Wood Technol..

[5] Izbicka J., Michalski J. (2006). Kompozyty i Laminaty—Tworzywa Stosowane w Technice. Pr. Inst. Elektrotechniki.

[6] Hu Y., Nakao T., Nakai T., Gu J., Wang F. (2005). Vibrational Properties of Wood Plastic Plywood. J. Wood Sci..

[7] Wojciechowska M., Kowaluk G. (2023). Waste Banana Peel Flour as a Filler in Plywood Binder. Ann. WULS For. Wood Technol..

[8] Wronka A., Kowaluk G. (2023). Upcycling of Wood Dust from Particleboard Recycling as a Filler in Lignocellulosic Layered Composite Technology. Materials.

[9] Yan Y., Wang J., Shen Z., Bi H., Shentu B. (2023). Flame Resistance and Bonding Performance of Plywood Fabricated by Guanidine Phosphate-Impregnated Veneers. Forests.

[10] Amini M.H.M., Hermawan A., Sulaiman N.S., Sobri S.A., Demirel G.K. (2022). Evaluation of Environmentally Friendly Plywood Made Using Glutaraldehyde Modified Starch As the Binder. Bull. Transilv. Univ. Brasov Ser. II For. Wood Ind. Agric. Food Eng..

[11] Walkiewicz J., Kawalerczyk J., Mirski R., Dziurka D. (2022). The Application of Various Bark Species as a Fillers for UF Resin in Plywood Manufacturing. Materials.

[12] Walkiewicz J., Kawalerczyk J., Mirski R., Szubert Z. (2023). The Tea Leaves As a Filler for Uf Resin Plywood Production. Wood Res..

[13] Auriga R., Gumowska A., Szymanowski K., Wronka A., Robles E., Ocipka P., Kowaluk G. (2020). Performance Properties of Plywood Composites Reinforced with Carbon Fibers. Compos. Struct..

[14] Liu Y., Guan M., Chen X., Zhang Y., Zhou M. (2019). Flexural Properties Evaluation of Carbon-Fiber Fabric Reinforced Poplar/Eucalyptus Composite Plywood Formwork. Compos. Struct..

[15] Bal B.C., Bektaş İ., Mengeloğlu F., Karakuş K., Ökkeş Demir H. (2015). Some Technological Properties of Poplar Plywood Panels Reinforced with Glass Fiber Fabric. Constr. Build. Mater..

[16] Jorda J., Kain G., Barbu M.C., Köll B., Petutschnigg A., Král P. (2022). Mechanical Properties of Cellulose and Flax Fiber Unidirectional Reinforced Plywood. Polymers.

[17] Król P., Borysiuk P., Mamiński M. (2019). Comparison of Methodologies for Acid Buffering Capacity Determination-Empirical Verification of Models. Appl. Sci..

[18] Kucukali Ozturk M., Venkataraman M., Mishra R. (2018). Influence of Structural Parameters on Thermal Performance of Polypropylene Nonwovens. Polym. Adv. Technol..

[19] Cusick G.E., Hearle J.W.S., Michie R.I.C., Peters R.H., Stevenson P.J. (1963). Physical Properties of Some Commercial Non-Woven Fabrics. J. Text. Inst. Proc..

[20] Wnorowska M., Dziurka D., Fierek A., Mrozek M., Borysiuk P., Hikiert M.A., Kowaluk G., Wnorowska M., Dziurka D., Fierek A., Mrozek M., Borysiuk P., Hikiert M.A., Kowaluk G. (2017). Przewodnik Po Płytach Drewnopochodnych. Wydanie II Poprawione.

[21] Yilmaz K.B., Sabuncuoglu B., Yildirim B., Silberschmidt V.V. (2020). A Brief Review on the Mechanical Behavior of Nonwoven Fabrics. J. Eng. Fiber. Fabr..

[22] Sadrolodabaee P., Claramunt J., Ardanuy M., de la Fuente A. (2021). Characterization of a Textile Waste Nonwoven Fabric Reinforced Cement Composite for Non-Structural Building Components. Constr. Build. Mater..

[23] Hasan K.M.F., Horváth P.G., Alpár T. (2021). Potential Fabric-Reinforced Composites: A Comprehensive Review. J. Mater. Sci..

[24] Patnaik P.K., Swain P.T.R., Mishra S.K., Purohit A., Biswas S. (2019). Recent Developments on Characterization of Needle-Punched Nonwoven Fabric Reinforced Polymer Composites—A Review. Mater. Today Proc..

[25] (1993). Wood-Based Panels. Determination of Modulus of Elasticity in Bending and of Bending Strength.

[26] (2011). Particleboards and Fibreboards. Determination of Resistance to Axial Withdrawal of Screws.

[27] (1993). Particleboards and Fibreboards. Determination of Tensile Strength Perpendicular to the Plane of the Board.

[28] (1993). Particleboards and Fiberboards. Determination of Swelling in Thickness after Immersion in Water.

[29] Dasiewicz J., Wronka A. (2023). Influence of the Use of Chestnut Starch as a Binder Filler in Plywood Technology. Ann. Warsaw Univ. Life Sci. SGGW For. Wood Technol..

[30] (2015). Plywood—Specification.

[31] Bal B.C., Bektaş I. (2014). Some Mechanical Properties of Plywood Produced from Eucalyptus, Beech, and Poplar Veneer. Maderas Cienc. Tecnol..

[32] Biadała T., Czarnecki R., Dukarska D. (2015). Attempt to Produce Flexible Plywood with Use of Europe Wood Species. Wood Res..

[33] Réh R., Igaz R., Krišt’ák L., Ružiak I., Gajtanska M., Božíková M., Kučerka M. (2019). Functionality of Beech Bark in Adhesive Mixtures Used in Plywood and Its Effect on the Stability Associated with Material Systems. Materials.

[34] Li W., Zhang Z., Zhou G., Leng W., Mei C. (2020). Understanding the Interaction between Bonding Strength and Strain Distribution of Plywood. Int. J. Adhes. Adhes..

[35] Maleki S., Kazemi Najafi S., Ebrahimi G., Ghofrani M. (2017). Withdrawal Resistance of Screws in Structural Composite Lumber Made of Poplar (Populus Deltoides). Constr. Build. Mater..

[36] Král P., Klímek P., Mishra P.K., Rademacher P., Wimmer R. (2014). Preparation and Characterization of Cork Layered Composite Plywood Boards. BioResources.

[37] Buddi T., Singh S.K., Nageswara Rao B. (2018). Optimum Process Parameters for Plywood Manufacturing Using Soya Meal Adhesive. Mater. Today Proc..

[38] Liu Y., Guan M. (2019). Selected Physical, Mechanical, and Insulation Properties of Carbon Fiber Fabric-Reinforced Composite Plywood for Carriage Floors. Eur. J. Wood Wood Prod..

[39] Bal B.C. (2017). Propriedades de Fixação de Parafusos e Pregos Em Painéis Compensados de Madeira Reforçados Com Tecido de Fibra de Vidro. Cerne.

[40] Daniłowska A., Kowaluk G. (2020). The Use of Coffee Bean Post-Extraction Residues as a Filler in Plywood Technology. Ann. WULS For. Wood Technol..

[41] Buzo A.L.S.C., Sugahara E.S., de Mello da Silva S.A., Morales E.A.M., dos A., Azambuja M. (2019). Painéis de Pínus e Bagaço de Cana Empregando-Se Dois Adesivos Para Uso Na Construção Civil. Ambient. Construído.

[42] Sugahara E.S., Da Silva S.A.M., Laura A., Buzo S.C., De Campos C.I., Morales E.A.M., Ferreira B.S., Azambuja M.D.A., Lahr F.A.R., Christoforo A.L. (2019). High-Density Particleboard Made from Agro-Industrial Waste and Different Adhesives. BioResources.

[43] Gao Q., Zhu Q., Guo Y., Yang C.Q. (2009). Formation of Highly Hydrophobic Surfaces on Cotton and Polyester Fabrics Using Silica Sol Nanoparticles and Nonfluorinated Alkylsilane. Ind. Eng. Chem. Res..

[44] Benkreif R., Brahmia F.Z., Csiha C. (2021). Influence of Moisture Content on the Contact Angle and Surface Tension Measured on Birch Wood Surfaces. Eur. J. Wood Wood Prod..

[45] Setter C., Zidanes U.L., de Novais Miranda E.H., Brito F.M.S., Mendes L.M., Junior J.B.G. (2021). Influence of Wood Species and Adhesive Type on the Performance of Multilaminated Plywood. Environ. Sci. Pollut. Res..

